# Visualization of Marek’s Disease Virus Genomes in Living Cells during Lytic Replication and Latency

**DOI:** 10.3390/v14020287

**Published:** 2022-01-29

**Authors:** Tereza Vychodil, Darren J. Wight, Mariana Nascimento, Fabian Jolmes, Thomas Korte, Andreas Herrmann, Benedikt B. Kaufer

**Affiliations:** 1 Institut für Virologie, Freie Universität Berlin, Robert von Ostertag-Straße 7-13, 14163 Berlin, Germany; tereza.vychodil@fu-berlin.de (T.V.); d.wight@fu-berlin.de (D.J.W.); m.nascimento@fu-berlin.de (M.N.); 2 Department of Biology, Molecular Biophysics, Humboldt-Universität zu Berlin, Invalidenstraße 42, 10115 Berlin, Germany; jolmes@picoquant.com (F.J.); thomas.korte@rz.hu-berlin.de (T.K.); andreas.herrmann@rz.hu-berlin.de (A.H.); 3 Institut für Chemie und Biochemie, Freie Universität Berlin, Altensteinstr. 23a, 14195 Berlin, Germany; 4 Veterinary Centre for Resistance Research (TZR), Freie Universität Berlin, 14163 Berlin, Germany

**Keywords:** Marek’s disease virus, live-cell genome visualization, lytic replication, T cells, latency, genome integration, TetO/TetR system

## Abstract

Visualization of the herpesvirus genomes during lytic replication and latency is mainly achieved by fluorescence in situ hybridization (FISH). Unfortunately, this technique cannot be used for the real-time detection of viral genome in living cells. To facilitate the visualization of the Marek’s disease virus (MDV) genome during all stages of the virus lifecycle, we took advantage of the well-established tetracycline operator/repressor (TetO/TetR) system. This system consists of a fluorescently labeled TetR (TetR-GFP) that specifically binds to an array of *tetO* sequences. This *tetO* repeat array was first inserted into the MDV genome (vTetO). Subsequently, we fused TetR-GFP via a P2a self-cleaving peptide to the C-terminus of the viral interleukin 8 (vIL8), which is expressed during lytic replication and latency. Upon reconstitution of this vTetO-TetR virus, fluorescently labeled replication compartments were detected in the nucleus during lytic replication. After validating the specificity of the observed signal, we used the system to visualize the genesis and mobility of the viral replication compartments. In addition, we assessed the infection of nuclei in syncytia as well as lytic replication and latency in T cells. Taken together, we established a system allowing us to track the MDV genome in living cells that can be applied to many other DNA viruses.

## 1. Introduction

Marek’s disease virus (MDV), also known as Gallid alphaherpesvirus 2 (GaHV-2), is a highly oncogenic herpesvirus that belongs to the genus Mardivirus. MDV infects chickens and causes neurological disorders, immunosuppression, paralysis, and deadly T cell lymphomas in various organs [1]. The virus enters the host through the respiratory tract where it infects macrophages and dendritic cells [2,3] that transport the virus to lymphoid organs. Here, this cell-associated virus is passed on to B and T cells in which it can replicate lytically [4,5,6]. In addition, MDV establishes latency predominantly in CD4+ T cells [7,8] and integrates its genome into the host telomeres [9,10]. During latency, only a few genes are expressed, including the major oncogene Meq (MDV005 and MDV076), splice variants of the viral chemokine vCXCL13 (aka. vIL-8; MDV003 and MDV078), [11,12] and the viral telomerase RNA [13,14]. Latently infected cells can also be transformed, resulting in the rapid formation of T cell lymphomas [15]. In addition, these cells can transport the virus to the feather follicle epithelia, where the virus replicates, is shed into the environment, and, thereby, spreads to naïve chickens [9].

Despite many years of research, many molecular processes involved in MDV replication and integration remain poorly understood. This is mostly due to the cell associated nature of the virus, its slow replication cycle, and the limited availability of tools. In recent years, viruses harboring fluorescent proteins have drastically expanded our knowledge on MDV replication and other processes during infection [16,17,18,19,20,21,22]. While virus proteins can be easily visualized in living infected cells by fusing them to, for example, green fluorescent protein (GFP), it remained impossible to visualize the virus genome in living cells. This genome visualization would provide valuable tools to assess the molecular processes including replication, integration, and latency.

In this study, we used the well-established tetracycline operator/repressor (*tetO*/TetR) system to visualize MDV genomes in living cells. The *tetO*/TetR system consists of a fluorescently labeled TetR protein (TetR-GFP) that specifically binds *tetO* repeat sequences as a dimer and, thereby, provides an increased fluorescent signal at the *tetO* insertion site [23]. This tool is commonly used in cell biology where cellular chromosome loci harboring *tetO* repeats are visualized to assess chromosome dynamics [24,25]. To monitor the MDV genome during infection, we inserted 112× *tetO* repeats into the virus genome using two-step Red-mediated mutagenesis [26]. TetR-GFP or TetR-mCherry were either expressed from the virus genome or stably expressed by the host cells. This system allowed us to visualize the MDV genome during lytic replication and in latently infected cells, providing important insights into the formation of replication compartments (RC), the structures in the nucleus in which the viral genome is replicated, and the infection of T cells.

## 2. Materials and Methods

### 2.1. Cells

Chicken embryo cells (CECs) were prepared from Valo specific-pathogen free (SPF) 11-day-old embryonated chicken eggs (Valo BioMedia, Osterholz-Scharmbeck, Germany), as described previously [27]. CECs were maintained in minimum essential medium (PAN Biotech, Aidenbach, Germany) supplemented with 10% fetal bovine serum (FBS, PAN Biotech) and 1% penicillin/streptomycin at 37 °C in a 5% CO_2_ environment. ESCDL-1, cell line derived from chicken embryonic stem cells [28] were maintained in DMEM Ham’s F12 (PAN Biotech) and supplemented with 10% FBS and 1% penicillin/streptomycin at 37 °C under a 5% CO_2_ atmosphere. The reticuloendotheliosis virus-transformed chicken T-cell line 855-19 was kindly provided by Prof. Thomas Göbel (Ludwig Maximilian University of Munich, Germany). Cells were maintained in RPMI (PAN Biotech) and supplemented with 10% FBS, 1% sodium pyruvate (PAN Biotech), 1% non-essential amino acids (Biochrom; Berlin, Germany), and 1% penicillin/streptomycin at 41 °C under a 5% CO_2_ atmosphere. All stable cell lines were confirmed by PCR to be mycoplasma-free.

### 2.2. Generation of Recombinant Viruses

The recombinant viruses harboring the *tetO*/TetR system components were generated based on a previously generated bacterial artificial chromosome (BAC) clone of the very virulent RB1B strain [29], in which most of the internal repeat short and long regions were deleted (ΔIR_L_ΔIR_S_) [15]. This deletion is rapidly restored upon reconstitution and facilitates a rapid manipulation of the repeat regions using two-step Red-mediated mutagenesis, as described previously [26,30]. First, transfer plasmids were generated that allowed the insertion of the components into the virus genome. To obtain the TetR-GFP transfer plasmid, the TetR-GFP cassette containing a nuclear localization signal (NLS) was amplified from the a p128tetR-GFP plasmid, kindly provided by Susan Gasser (Friedrich Miescher Institute, Switzerland) [25], and cloned into pcDNA3.1 using BamHI and EcoRI. The kanamycin selection cassette (kana_I-SceI) was amplified from pEPkan-S1, homologue sequences for its removal inserted via the primer overhangs and cloned into pcDNA3.1_TetR-GFP. This mutagenesis cassette was subsequently used to insert TetR-GFP (i) after the strong HSV-1 thymidine kinase (TK) promoter within the mini-F cassette (vTetR) or (ii) fused to the C-terminus of vCXCL13 via a P2A self-cleaving peptide (vTetO/TetR), resulting in expression of TetR-GFP and vCXCL13 as separate proteins, using two-step Red-mediated mutagenesis, as described previously [26,30].

**Figure 1:**
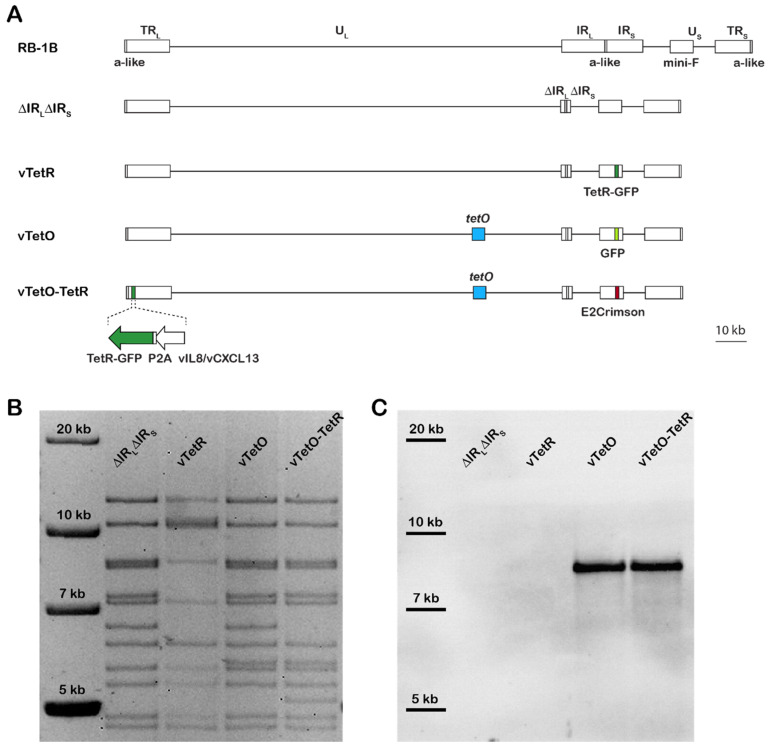
Generation of recombinant viruses. (**A**) Overview of the MDV genome and the recombinant BAC clones. The MDV genome consists of a unique long and short region (U_L_ and U_S_) flanked by the terminal repeat long and short (TR_L_, TR_S_) and the internal repeat long and short (IR_L_, IR_S_). The a–like sequences harboring the cleavage and packaging signals are indicated. The recombinant viruses were generated based in the ΔIR_L_ΔIR_S_ BAC lacking most of the IR_L_ and IR_S_ region, which is rapidly restored upon reconstitution. TetR-GFP is depicted in dark green, *tetO* sequence in blue, GFP in light green, and E2-Crimson in red; (**B**) RFLP of indicated recombinant BAC genomes using NdeI; (**C**) Southern blot of the same RFLP gel using a specific TetO probe to confirm the presence and length of the *tetO* sequences in the virus genome.

To obtain the TetO transfer plasmid, the 112× *tetO* repeats array (4.5 kbp) of pRS306tet02x112 (Susan Gasser, Friedrich Miescher Institute, Switzerland) was cloned into a vector containing long homologue sequences of UL45 and UL46 (MDV058 and MDV059, respectively). The *tetO* sequence was inserted between UL45 and UL46 using BamHI and BglII. The kana_I-SceI cassette with homologue sequences for its removal were inserted into the UL45 homologue arm. This mutagenesis cassette was subsequently used to insert *tetO* between UL45 and UL46 of the virus genome using two-step Red-mediated mutagenesis (Figure 1A, vTetO and vTetO-TetR). This UL45/UL46 was chosen as it previously allowed the insertion of foreign genes without affecting the MDV replication [16]. In addition, either GFP or E2-Crimson, driven by the HSV-1 TK promoter, was inserted in the mini-F vector to facilitate the detection of infected cells (for vTetO and vTetO-TetR, respectively).

All recombinant virus genomes were confirmed by restriction fragment length polymorphism (RFLP) analyses, Southern blotting, Sanger sequencing, and next-generation sequencing (NGS; Illumina MiSeq) of the entire virus genome. All primers used for cloning and mutagenesis can be found in Table 1.

### 2.3. Southern Blotting

To confirm the insertion of the *tetO* repeats, DNA of BAC clones were digested with indicated enzymes and separated on a 0.8% agarose gel. DNA in the agarose gel was denatured (0.5 M NaOH, 1.5 M NaCl) and transferred to a positively charged nylon membrane (Immobilon-NY+, Merck Millipore, Darmstadt, Germany) and incubated with TetO DIG-labeled probe (Table 1), as described previously [9]. *TetO* repeats were detected using an anti-DIG alkaline phosphatase-labeled antibody (Roche GmbH, Mannheim, Germany).

### 2.4. Illumina MiSeq Sequencing

BAC DNA of the vTetO and vTetO-TetR clones were sequenced using Illumina MiSeq (Illumina, San Diego, CA, USA). Sequencing libraries were prepared using the NEBNext^®^ Ultra™ II DNA Library Prep Kit for Illumina^®^ (New England Biolabs, Ipswich, MA, USA). The generated Illumina reads were processed with Trimmomatic v.0.39 [31] and mapped against the RB-1B TetO (GenBank accession no. OM350391) and RB-1B TetO TetR-GFP (GenBank accession no. OM350392) GenBank references, respectively, using the Burrows-Wheeler aligner v.0.7.17 [32]. Single nucleotide polymorphisms (SNPs), insertions, and deletions were assessed with FreeBayes v.1.1.0-333 [33]. Data were merged by position and mutation using R v.3.2.3; the coverage was additionally assessed and generated using Geneious R11 software.

### 2.5. Plaque Size Assay and Growth Kinetics

Recombinant viruses were reconstituted using calcium-phosphate transfection of CECs and ESCDL-1 with respective BAC clones, as described previously [34]. Spread of recombinant viruses and replication properties in vitro were determined by plaque size assay and multi-step growth kinetics. For plaque size assays, one million CECs were infected with 100 plaque forming units (PFU) of each virus (passage 5 and 7) and the area of 50 randomly selected plaques were measured using ImageJ (https://imagej.nih.gov/ij/, accessed on 4 June 2020) and normalized against ΔIR_L_ΔIR_S_. In addition, plaques were also measured 6 days after BAC DNA transfection. For multi-step growth kinetics, one million CECs were infected with 100 PFU in 6-well plates per virus and cultured for 6 days. Every day, one well per plate was harvested and stored at −80 °C. After 6 days, DNA of all samples was extracted using Zymo Quick DNA Viral kit (Zymo Research Europe GmbH, Freiburg, Germany), according to the manufacturer’s instructions. MDV genome copy numbers were determined by qPCR using specific primers and probe for the MDV polymerase (MDV043, UL30). UL30 copy numbers were normalized against the chicken inducible nitric oxide synthase (iNOS), as described previously [35]. Primers and probes used for qPCR are listed in Table 1.

### 2.6. Assessment of TetO Stability by Nanopore Sequencing

To determine if the array of *tetO* repeats is stably maintained in the virus, extrachromosomal DNA of CECs infected with higher passages of vTetO and vTetO-TetR (passage 9) was extracted using Hirt extraction, as described previously [36,37]. Briefly, infected cells from 150 mm dishes were trypsinized and pelleted by centrifugation at 4 °C and 800× *g* for 5 min. The pellet was washed with ice-cold PBS, centrifuged again, resuspended in 400 µL of Hirt lysis buffer (10 mM Tris-HCl, 20 mM EDTA, 1.2% SDS, pH 8.0) and incubated for 20 min at room temperature. Next, 200 µL of 5 M NaCl was added and incubated at 4 °C for ≥16 h followed by centrifugation at 4 °C and 15.000× *g* for 30 min to pellet proteins and chromosomes. The extrachromosomal DNA in the supernatants was then purified by phenol-chloroform extraction, precipitated with isopropanol, and washed with ethanol. The obtained viral genomes were used for nanopore sequencing. Nanopore libraries were prepared using the SQK-LSK110 Ligation Sequencing Kit (Oxford Nanopore Technologies, Oxford, UK) and sequenced using a Flongle flow cell (FLO-FLG001, Oxford Nanopore Technologies, Oxford, UK) on a MinION sequencer (MK-1B, Oxford Nanopore Technologies, Oxford, UK). The resulting Nanopore reads were mapped against the RB-1B TetO and RB-1B TetO TetR-GFP GenBank references using Minimap2 [38] embedded on Nanopore’s MinKNOW GUI. Alignments were viewed using IGV Web App [39,40].

### 2.7. Generation of T Cell Line Stably Expressing TetR-mCherry

To generate a cell line stably expressing TetR-mCherry, the 855-19 chicken T-cell line was transduced with the pQCXIN-TetR-mCherry retroviral system (kindly provided by Tom Misteli from National Cancer Institute, Addgene plasmid #59417) [41] and selected in the presence of 1200 µg/mL Geneticin (Roth, Karlsruhe, Germany). The newly generated cell line was confirmed to be mycoplasma-free by PCR.

### 2.8. Wide-Field Microscopy

To investigate if the recombinant viruses efficiently express TetR-GFP, we infected CECs and ESCDL-1 cells with vTetR and/or vTetO-TetR. At 4 dpi, we stained the nuclei with Hoechst 33342 (Invitrogen, Carlsbad, CA, USA) for 30 min and fixed the cells with 4% paraformaldehyde (PFA). Wide-field images of infected cells were taken using Axio Imager M1 (Zeiss, Oberkochen, Germany) equipped with Axio Cam MRm camera (Zeiss) with a 100×/1.4 Oil Plan-Apochromat objective (Zeiss). Images were further processed in ImageJ.

### 2.9. Confocal Microscopy and Live-Cell Imaging

For live imaging, cells were grown in pre-coated µ-Slide ibiTreat plates with polymer coverslip (Ibidi, Gräfelfing, Germany). Cells were maintained in medium supplemented with 0.2 M HEPES (Roth). 855-19 cells were also immobilized in 0.25% low melting-point agarose (Gibco BRL, Carlsbad, CA, USA). Nuclei were stained with Hoechst 33342 for 30 min. To confirm TetR binding specificity, cells were incubated with 2 µg/mL tetracycline (Roth) for 1 h.

Live microscopy was performed using (i) a VisiScope spinning disk confocal system (Visitron Systems, Puchheim, Germany, CSU-W1; Yokogawa, Tokyo, Japan) built on a Nikon Eclipse Ti inverted microscope equipped with an iXon Ultra 888 EMCCD camera and an OkoLab gas and temperature controller (OkoLab, Ottaviano, Italy) to maintain a 5% CO_2_ atmosphere at 37 °C or (ii) a FluoView1000 inverted confocal microscope (Olympus, Tokyo, Japan). If not indicated otherwise, the VisiScope spinning disc confocal microscope was used. Images were captured with (i) 60×/1.4 and 100×/1.45 Oil Plan-Apo objectives (Nikon) using VisiView software (v.4.3.0.6; Visitron Systems, Puchheim, Germany) or (ii) 60× water immersion objective (UPlanSApo) using Olympus FluoView software (v 4.02; Olympus, Tokyo, Japan). Z-step size between focal planes was 0.5 µm and final 2D images are visualized as maximum intensity projection. All images and videos were processed with ImageJ software.

### 2.10. Lymphocyte Infection

To infect 855-19 T-cells expressing TetR-mCherry, one million CECs were infected with 10,000 PFU of vTetO. After 6 days, CECs were overlaid with one million T-cells and incubated together for 24 h in RPMI (PAN Biotech) supplemented with 10% FBS, 1% sodium pyruvate (PAN Biotech), 1% non-essential amino acids (Biochrom, Berlin, Germany), and 1% penicillin/streptomycin at 41 °C under a 5% CO_2_ atmosphere. The next day, T-cells were carefully harvested and the infection rate was determined by flow cytometry detecting eGFP expressed in infected cells using a CytoFLEX S system (Beckman Coulter, Krefeld, Germany). Infected T-cells were maintained in culture for 14 days to allow the virus to establish latency and integrate its genome into host chromosomes. Images were taken 1, 3, and 14 days post infection (dpi) using the VisiScope spinning disc confocal microscope.

### 2.11. Fluorescence In Situ Hybridization (FISH)

Interphase nuclei were prepared from infected 855-19 T-cells 3 dpi (lytic) and 14 dpi (latent), as described previously [42]. Briefly, MDV genomes were detected using a set of PCR-based MDV probes and visualized using Cy3 Streptavidin (1:1000 dilution; GE Healthcare, Munich, Germany) [43]. Images of interphases were taken using Axio Imager M1 (Zeiss) and analyzed with the ImageJ software.

### 2.12. Statistical Analysis

Statistical analyses were performed using GraphPad Prism software v.8.0.2. Data were considered significantly different for *p* values of ≤0.05. Description of all applied statistical tests can be found in the respective figure legends. If not noted differently, all experiments were repeated at least three independent times.

## 3. Results

### 3.1. Generation of Recombinant Viruses

To visualize MDV genomes in living cells, we generated recombinant viruses that harbor the *tetO*-repeats using the very virulent RB-1B strain [35,44]. The *tetO* repeat cassette (4.5 kbp) was inserted in between UL45 and UL46, a locus well established for the insertion of foreign genes without affecting virus replication [16]. The TetR fused to a fluorescence protein and a nuclear localization signal (NLS) and was then either expressed by the virus or the cells depending on the application. To exclude that the TetR protein alone forms unspecific aggregates in the nucleus of chicken cells, we generated a virus expressing high levels of TetR-GFP driven by the strong TK promoter in the mini-F in the absence of *tetO* (vTetR). Since we observed that high expression levels of TetR-GFP driven by the TK promoter reduced the signal to noise ratio, we tested various loci for the insertion of the protein. The optimal expression was achieved by fusing TetR-GFP via a P2A self-cleaving peptide to the C-terminus of vCXCL13 (aka. vIL-8). This facilitated TetR-GFP expression during both lytic replication and latency at optimal levels. To identify infected cells, we inserted the GFP into the virus only containing *tetO* (vTetO) and the far-red protein E2-Crimson into the vTetO-TetR double insertion virus (vTetO-TetR) into the mini-F (Figure 1A). The resulting clones were analyzed by RFLP (Figure 1B) and Sanger sequencing. The presence and length of the *tetO* repeats in the viral genome were confirmed by Southern blotting (Figure 1C). The specific TetO probe detected the fragment with the expected size in both vTetO and vTetO-TetR, indicating that the full length *tetO* cassette was inserted during mutagenesis. Illumina MiSeq next generation sequencing was performed and confirmed that no additional mutations are present in the recombinant virus genomes.

### 3.2. Characterization of Replication Properties

**Figure 2:**
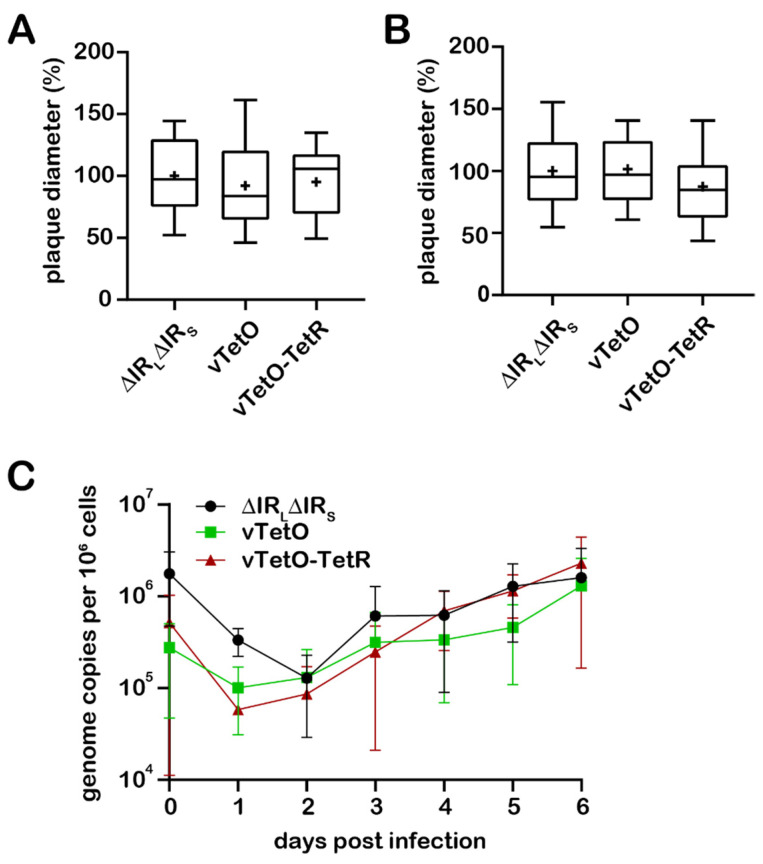
In vitro characterization of the recombinant viruses. Plaque size assays after (**A**) transfection and (**B**) infection with passaged viruses, as indicated. Data are shown as means of three independent experiments with means (+), medians (line within the bar), and standard deviations (*p* > 0.05, one-way analysis of variance (ANOVA) Dunnett’s test). (**C**) Representative multi-step growth kinetics of indicated viruses with standard deviations (*p* > 0.05, one-way analysis of variance (ANOVA) Kruskal–Wallis test).

To examine if the insertion of *tetO* and TetR-GFP affects virus replication, we performed plaque size assays and multi-step growth kinetics. No significant difference was observed in plaque size assays performed after transfection compared to the parental virus (Figure 2A). Similarly, no significant difference was detected in plaque size assays upon serial passaging of the viruses (passage 7; Figure 2B). These results were confirmed by multi-step growth kinetic analyses (Figure 2C), highlighting that insertion of *tetO* and TetR-GFP has no significant impact in MDV replication. To further investigate the stability of the *tetO* repeats in the viral genome, we isolated extrachromosomal DNA from CECs infected with high-passaged virus (passage 9) and performed nanopore sequencing on the MDV genomes. The results from nanopore sequencing demonstrated that *tetO* is stably maintained in the UL45-UL46 locus in both vTetO and vTetO-TetR, highlighting that the system could even be used with high passage stocks.

### 3.3. Visualization of the Virus Genome during Lytic Replication and Specificity of TetR Binding

**Figure 3:**
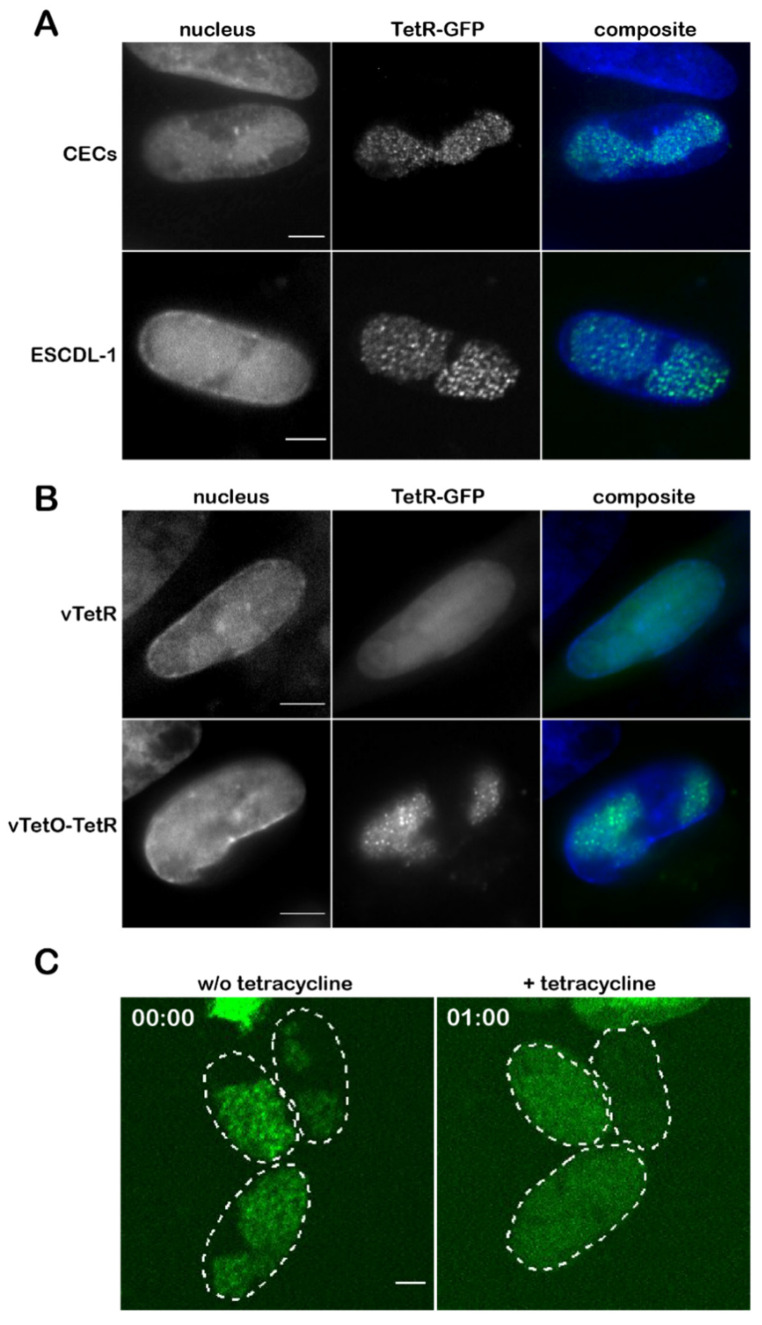
Detection of virus genomes in infected cells. (**A**) CECs and ESCDL-1 cells were infected with vTetO-TetR, fixed with 4% PFA at 4 dpi, and imaged with an Axio Imager M1 using a 100× oil objective. Scale bar corresponds to 5 µm; (**B**) CECs were infected with either vTetR or vTetO-TetR, fixed at 4 dpi using VisiScope spinning-disk confocal microscope with a 100× oil objective. Scale bar corresponds to 5 µm; (**C**) CECs infected with vTetO-TetR were treated with tetracycline. Images are shown before (**left** panel) and 1 h after the addition of tetracycline (**right** panel). Image stacks of nine focal planes were captured with a z-step size of 0.5 µm and displayed as maximum-intensity projection (MIP). The perimeter of the nuclei are depicted as dotted lines. Time (in hh:mm) and scale bar (5 µm) are indicated.

To visualize MDV genomes during lytic replication, we infected primary CECs and the chicken ESCDL-1 cell line with 100 pfu of vTetO-TetR, counterstained with Hoechst 33342 at four dpi imaged the cells. In both CECs and ESCDL-1, we consistently observed one to two replication compartments (RCs) per nucleus that were visually separated from each other (Figure 3A). To ensure that TetR alone does not establish unspecific aggregates, we infected CECs with vTetR and vTetO-TetR. In cells infected with vTetR we observed a uniform TetR-GFP signal in the nucleus. In contrast, in cells infected with vTetO-TetR we detected specific signal for the virus genome, forming RCs within the nucleus (Figure 3B). To further validate the specificity of the TetO/TetR signal, we used tetracycline to induce a conformational change of the TetR DNA-binding domain, resulting in the dissociation of TetR-GFP from the *tetO* repeats [45]. CECs were infected with vTetO-TetR for 4 days and imaged before and after the addition of tetracycline. Neighboring infected cells harbored RCs in their nucleus before adding tetracycline. Upon addition of the drug, TetR-GFP dissociates and the specific signal pattern was lost, highlighting that the observed genome staining is highly specific (Figure 3C).

### 3.4. Genesis and Mobility of Replication Compartments

**Figure 4:**
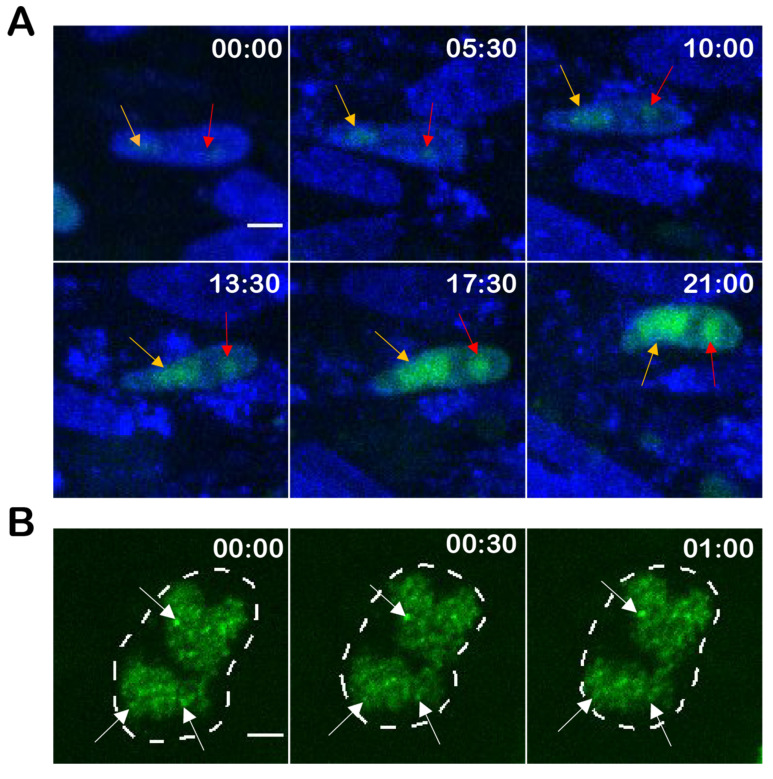
Genesis and mobility of replication compartments (RC). (**A**) The development of RCs in infected cells were monitored for 21 h. Sequential images were taken with spinning-disk confocal microscope using a 60× water immersion objective every ten minutes. Multiple Z-stacks were processed as maximum-intensity projection (MIP). RC 1 and RC 2 are indicated with an orange arrow and red arrow, respectively. Nuclei are shown in blue, TetR-GFP in green. Time in hh:mm, scale bar 5 µm; (**B**) the mobility of the RCs in infected cells was monitored for 1 h. Stacks of 15 focal planes with a z-step size of 0.5 µm were captured at 5 min intervals using a VisiScope microscope. Images represent the MIP of each time point. Nucleus contour is depicted as dotted line; white arrows indicate non-moving dotted structures. Time in hh:mm, scale bar 3 µm.

Next, we investigated the formation of the RCs in infected cells. We infected CECs with vTetO-TetR for 4 days, counterstained the nuclei with Hoechst 33342, and monitored the development of RCs in newly infected cells via live cell imaging for 21 h. Separated RCs in the nuclei were observed early in infection (Figure 4A). During the 21 h period, the RCs gradually increased in size (RC 1 from 6.6 µm^2^ to 20.5 µm^2^; RC 2 from 1.5 µm^2^ to 5.7 µm^2^).

Based on the previous findings that RCs have properties of phase-separated condensates (liquid-liquid phase separation) and proteins move freely within RCs [46], we set out to investigate the mobility of the viral DNA in these structures. At 4 dpi, we tracked infected nuclei for 1 h and captured stacks of 15 focal planes and analyzed the maximum-intensity projection (MIP) (Figure 4B) and rendered 3D images (Supplementary Video S1) of a representative nucleus. Although the nuclei themselves were moving during the imaging period, we observed that the shape of the RCs did not change and many of the dotted structures stayed in the same place suggesting that the viral DNA is rather immotile.

### 3.5. Detection of Both Infected and Uninfected Nuclei inside A Syncytium

**Figure 5:**
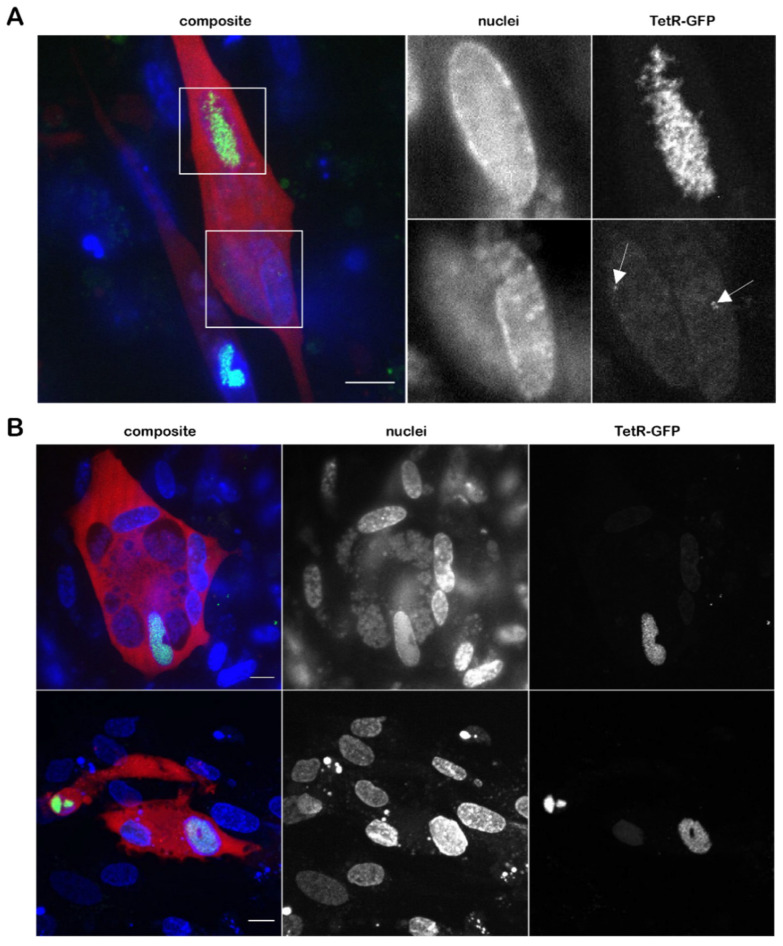
MDV can induce syncytia in CECs but replication is only detected in some of the nuclei. (**A**) Nuclei within a syncytium with varying TetR-GFP intensities. Image stacks of 21 focal planes with a z-step size of 0.5 µm are displayed as MIP. Nuclei were stained with Hoechst 33342 for 30 min and captured using WF-DAPI. Single or few MDV genomes are indicated by white arrows; (**B**) representative images of MDV syncytia. E2-Crimson in cytoplasm in red, nuclei in blue, and TetR-GFP in green. Scale bar 10 µm.

MDV has been previously shown to induce syncytia in duck embryo fibroblasts (DEF) [47,48]. Using our vTetO-TetR expressing E2-Crimson in the cytoplasm, we frequently observed that MDV can also induce syncytia in infected primary CECs. We, therefore, infected CECs with vTetO-TetR to assess if all nuclei were infected and showed replication compartments. Nuclei of the cells were stained with Hoechst 33342 and we imaged the cells using a spinning-disk confocal microscope at 4 dpi. Interestingly, we observed that some nuclei harbor RCs while others did not. In some nuclei, single dots were detected (Figure 5A), suggesting that only one or few viral genomes were present. In other syncytia, no virus signal was detected in some of the nuclei (Figure 5B), indicating that MDV does not replicate its genome in all nuclei of a syncytium.

### 3.6. Visualization of Viral Genomes during Lytic Replication and Latency in T Cells

**Figure 6:**
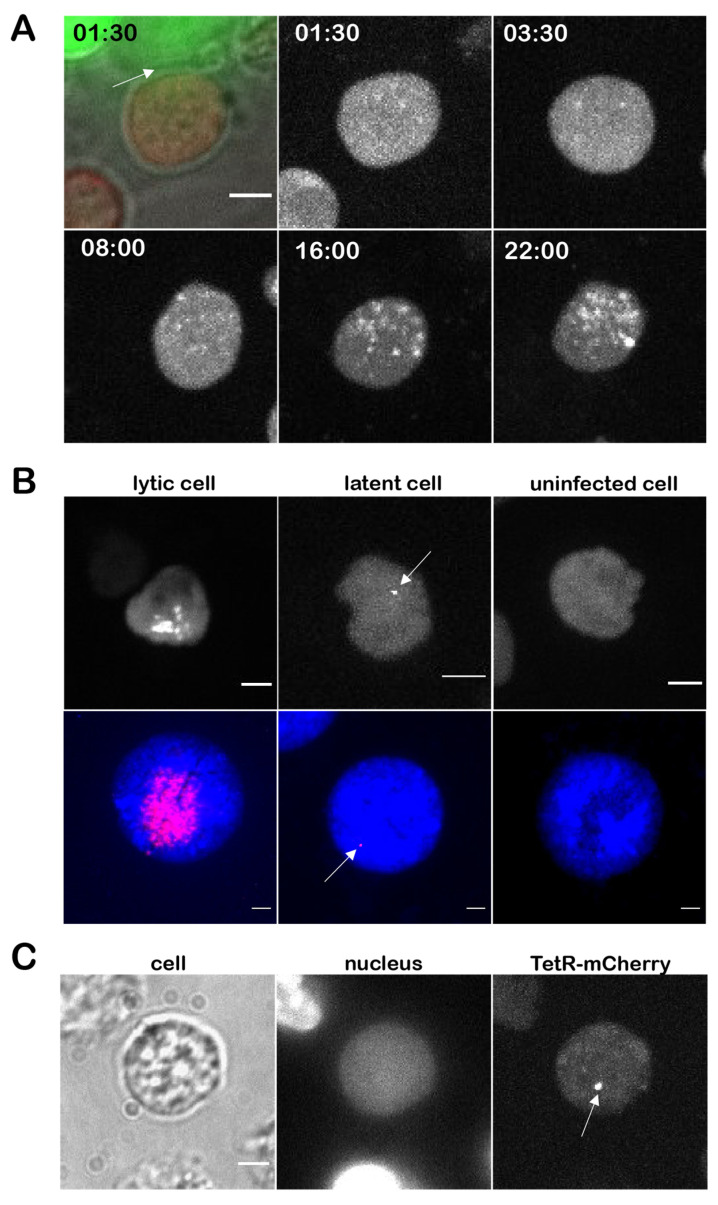
Lytic replication and latency in T cells. (**A**) 855-19 T cells stably expressing TetR-mCherry (multi-color image) were seeded on an infected CECs monolayer. The site of contact between infected CEC (green) and uninfected T cell (red) is shown with a white arrow. Time course images are shown as maximum intensity projections of the red channel (TetR-mCherry). Images were taken using a 60× Oil objective. 13 focal planes with a z-stack size of 1.7 µm were imaged every ten minutes for 21 h. Time in hh:mm, scale bar 3 µm (**B**) representative images of 855-19 T cells expressing TetR-mCherry three days after virus infection (upper line) in comparison with FISH images (lower line; DAPI in blue, viral DNA in red). Live cell images are shown as maximum intensity projection of z-stacks in the red channel. Arrows highlight single dots in the nucleus of latently infected cells. Scale bar 3 µm; (**C**) representative image of infected T cells at 14 dpi. One specific dot (arrow) was detected in maximum-intensity projection of multiple focal planes in TetR-mCherry.

To assess lytic replication and latency in T cells, we co-seeded CECs highly infected with vTetO with uninfected 855-19 T cells stably expressing TetR-mCherry. Imaging started after the T cells settled on the infected CEC monolayer. Cells were imaged using the spinning-disk confocal microscope every ten minutes for 21 h. DNA replication was detectable at 8 hpi and gradually increased until 22 hpi (Figure 6A). Intriguingly, we observed smaller and sparse RCs in T cells when compared with CECs and ESCDL-1 cells, indicating that MDV replication in T cells may differ.

To further investigate infection and the establishment of latency in T cells, vTetO infected lymphocytes were harvested at 24 hpi and maintained in cell culture for 14 days. At 3 dpi, few lytically infected cells were still observed (Figure 6B). Intriguingly, we also detected cells with a single dot in the nucleus (white arrow) after 3 dpi (Figure 6B). These pictures were confirmed with FISH preparations of infected cells and are consistent with recently published work done on 885-19 T cells [43]. Moreover, to ascertain that this single bright staining represents a latent virus genome, we analyzed the infected T cells also 14 dpi when no replication was observed. Single specific spots were observed during the latent phase upon infection of these T cells (Figure 6C, white arrow), highlighting that our system is sensitive enough to detect these latent virus genomes.

## 4. Discussion

The visualization of viral genomes in living cells has an immense potential in research on a broad range of processes including antiviral responses to the genome, viral replication, and genome maintenance in latently infected cells. Most processes involving the virus genome during MDV infection remain poorly understood. Therefore, we set out to develop a system that facilitates visualization of the MDV genome in living cells.

To visualize MDV genomes, we inserted the *tetO* repeats into the MDV genome between UL45 and UL46 genes (vTetO). Plaque size assays and multiple step growth kinetics revealed that insertion of the *tetO* repeats into this site does not affect MDV replication and cell-to-cell spread in culture. This was not surprising as this locus has been previously successfully used (e.g., for the insertion of a GFP expression cassette) [16]. Southern blotting confirmed the expected size of the *tetO* repeats and Illumina MiSeq NGS ensured that no additional SNPs were present in the recombinant viruses. More importantly, nanopore sequencing confirmed that the *tetO* repeats in the MDV genome are stable for at least nine passages, highlighting that the system could even be used with high passage viruses and for long-term analyses.

Next, we expressed the fluorescently tagged TetR either from the virus genome or from a plasmid stably maintained in the target cell. To ensure the reliability of the *tetO*/TetR system, we validated the specificity of the TetR staining by two independent approaches. First, we confirmed that overexpression of TetR-GFP driven by the strong TK promoter does not cause unspecific staining in the absence of *tetO*, for example, due to the formation of aggregates in the nucleus. In addition, we validated the TetR specificity via the addition of tetracycline, resulting in a conformational change of the TetR DNA-binding domain [45]. This resulted in the expected release of the TetR-GFP from the *tetO* repeats present in the virus and resulted in a diffuse staining observed in the absence of *tetO* (Figure 3C). Although we detected even distribution of TetR-GFP (Figure 3B) in the entire nucleus, the amount of TetR expressed from the TK-promoter was too high to be able to detect single viral genomes. In addition, most of the viral genes (including the TK promoter in the mini-F) are silenced during latency. Therefore, we decided to fuse TetR-GFP via a P2a self-cleaving peptide to the C-terminus of vCXCL13 in vTetO (Figure 1A), as splice variants containing the last exon (exon 3) are expressed during both lytic replication and latency at moderate levels [12,49].

Using the optimized *tetO*/TetR system, we could efficiently visualize RCs in lytically infected primary cells and cell lines (Figure 3A and Figure 5A). We could also observe the establishment and growth of the RCs using live-cell microscopy. In previous studies on HSV-1, it was shown that the vast majority of RCs are initiated from a single incoming viral genome at distinct sites in the nucleus [50,51]. Due to the strictly cell-associated nature of MDV, we could not perfectly time the infection, but could observe a similar phenotype. Our results indicate that it takes MDV RCs about 24 h to reach their full size upon entry into the nucleus. In addition, the observed one to two RCs within a nucleus possessed a different size and shape, although they were gradually growing (Figure 4A). In comparison, HSV-1 infected cells with a multiplicity of infection of 20 commonly harbor three to five RCs per nucleus depending on the cell line [52]. In T cells, MDV establishes usually just a diffuse RC that differs from the ones seen in fibroblasts. This could be due to the smaller size of T cell nuclei, their previously reported rigidity, and compact nature [53,54,55]. Our system, thereby, revealed a cell type dependent difference between the RCs.

Based on previous studies with HSV-1 [46,56], it is thought that viral genomes are phase-separated from chromosomal DNA and have the properties of liquid-liquid phase-separated condensates. It was shown that proteins can freely diffuse through RC, whereas the viral genomes appear to be much slower. However, these studies were done using HSV-1 amplicon plasmids that do not reflect the entire complexity of replicating herpesviruses [46,57]. Here, we observed that, although the infected cells and nuclei moved during the recording period, we detected only minimal changes in the shape of RC and position of the dotted structures within the RC (Figure 4B). This could be explained by the cohesion of concatemeric viral genomes during branched rolling circle replication, whereas proteins can freely diffuse through the RCs. We will assess the interaction of proteins with the MDV genome in this context using our MDV system in future studies.

Using the vTetO-TetR system, we observed that MDV can induce fusion of cultured CECs, resulting in the formation of syncytia. We examined this CPE, which was previously only reported in DEFs, and observed that some nuclei within a syncytium do not possess RC (Figure 5A). We noticed a weak but diffuse TetR-GFP signal in those nuclei, indicating that the fusion protein was, to some extent, transported into the uninfected nuclei within the syncytium due to its NLS.

To shed more light on the course of infection in T cells and the establishment of latency, we set out to explore the behavior of viral genomes early in infection. Since almost all MDV genes are silenced during latency, we generated T cell line stably expressing TetR-mCherry independent of the virus life cycle (Figure 6B). Replication foci were detected around 8 hpi that increased over the course of time (Figure 6A), indicating that lytic replication progresses in some of the T cells, as shown previously [4,5,6]. After 14 dpi we were able to detect single bright dots in the nucleus, as detected by FISH. This is consistent with previous studies that found only one or few integration sites in primary T cells and T cell lines latently infected in vitro [6,43], while multiple integration sites were detected in MDV-induced tumor cells [9,10]. Since no replication was detected at this time point, these most likely correspond to latent virus genomes (Figure 6B,C). Comparable signals were found in infected T cells already at 3 dpi, suggesting that MDV can enter in the quiescent stage early after infection. We will expand on these observations, especially in the context of MDV integration in the future.

Taken together, we established a system that facilitates the visualization of MDV genomes in living cells. This system provided exciting insights into the virus live cycle, including the number of RCs in different cell types, the establishment and expansion of RCs, the formation of syncytia, and the infection of T cells resulting in either lytic replication or latency. This *tetO*-TetR-based system, established in this study, will contribute to our understanding of MDV, replication, genome integration, and the establishment of latency in future studies.

## Figures and Tables

**Table 1:** Oligonucleotide sequences used in this study.

Construct/Steps	Direction	Sequence (5′–3′)
pCDNA3.1 TetR-GFP	for	TAGATGAGCTCGGATCCATGCCAAAGAAGAAGCGTAAG
	rev	GATGGATATCTGCAGAATTCTCATCCCATGCCATTGGT
TetR-GFP-kana transfer	for	TACAAGACACGTGCTGAAGTCAAGTTTGAAGGTAGGGATAACAGGGTAATCGATTT
	rev	ACTTCAGCACGTGTCTTGTAGTTCCCGTCATCGCCAGTGTTACAACCAATTAACC
TetR-GFP in mini-F (vTetR)	ep for	TTAAGGTGACACGCGCGGCCTCGAACACAGCTGCAGGCCGATGTACGGGCCAGATATACG
	ep rev	CGTCGACCCGGGTACCTCTAGATCCGCTAGCGCTTTATGTCTTCCCAATCCTCCCC
P2A-TetR-GFP into vIL-8/vCXCL13	ep for	ATTGAGCCCACACCTCCTACTATTGGTTCCCATATCTGTCTTGGTTCCGGAGCCACGAACTTCTCTCTGTTAAAGCAAGCAGGAGACGTGGAAGAAAACCCCGGTCCTATGCCAAAGAAGAAGCGTAAG
	ep rev	AAAGTGCCTTCTTTTAATTACAGGAGGTAGCAATTAATCATCCCATGCCATTGGTAATCC
TetO-kana transfer	for	GACAGTAGATCTACCTGTATACTACCCACCATTGTAGGGATAACAGGGTAATCGATTT
	rev	ACAGGTAGATCTACTGTCCCGTAGTCTAAATATGCCAGTGTTACAACCAATTAACC
eGFP in mini-F	ep for	GGTGACACGCGCGGCCTCGAACACAGCTGCAGGCCATGGTGAGCAAGGGCGAGG
	ep rev	CGTCGACCCGGGTACCTCTAGATCCGCTAGCGCTTTACTTGTACAGCTCGTCCATGCC
E2-Crimson into mini-F	ep for	TGCCCTTGCTAGGGTTCTTCACACGAGCCTCGCCTTATTAAATGGGCTCCGGTGCCCGTC
	ep rev	CCCGAGGCCTCGTGGGGCACCTATTTGCGCGGAGGAAGGCCCATAGAGCCCGGGCCATC
TetO DIG-probe		DIG-TCCCTATCAGTCATAGAGAAAAGTGAAAGTCGAGTTTACCAC
iNOS (qPCR)	for	GAGTGGTTTAAGGAGTTGGATCTGA
	rev	TTCCAGACCTCCCACCTCAA
	probe	FAM-CTCTGCCTGCTGTTGCCAACATGC-TAMRA
UL30 (qPCR)	for	AAGCGGAATCGGTTTACAAG
	rev	GGAGTTGCTGTTAGAATACGGA
	probe	FAM-TCGACGAGTTTCTTCCTCCTCGTTG-TAMRA
Mycoplasma test	for	GGGAGCAAACAGGATTAGATACCCT
	rev	TGCACCATCTGTCACTCTGTTAACCTC

ep, en passant mutagenesis primer; for, forward primer; rev, reverse primer; FAM, 6-carboxyfluorescein; TAMRA, 6-carboxytetramethylrhodamine; DIG, Digoxigenin.

## Data Availability

Not applicable.

## Supplementary Materials

The following supporting information can be downloaded at: https://www.mdpi.com/article/10.3390/v14020287/s1, Video S1: 3D image of RC in nucleus.
